# Modulation of macrophages by biophysical cues in health and beyond

**DOI:** 10.1093/discim/kyad013

**Published:** 2023-08-10

**Authors:** Heather M Wilson

**Affiliations:** School of Medicine, Medical Sciences and Nutrition, Institute of Medical Sciences, University of Aberdeen, Aberdeen, UK

**Keywords:** macrophages, mechanosensing, mechanotransduction, extracellular matrix, biophysical cues

## Abstract

Macrophages play a key role in tissue development and homeostasis, innate immune defence against microbes or tumours, and restoring homeostasis through tissue regeneration following infection or injury. The ability to adopt such diverse functions is due to their heterogeneous nature, which is driven largely by their developmental origin and their response to signals they encounter from the microenvironment. The most well-characterized signals driving macrophage phenotype and function are biochemical and metabolic. However, the way macrophages sense and respond to their extracellular biophysical environment is becoming increasingly recognized in the field of mechano-immunology. These biophysical cues can be signals from tissue components, such as the composition and charge of extracellular matrix or topography, elasticity, and stiffness of the tissue surrounding cells; and mechanical forces such as shear stress or stretch. Macrophages are important in determining whether a disease resolves or becomes chronic. Ageing and diseases such as cancer or fibrotic disorders are associated with significant changes in the tissue biophysical environment, and this provides signals that integrate with those from biochemical and metabolic stimuli to ultimately dictate the overall function of macrophages. This review provides a brief overview of macrophage polarization, followed by a selection of commonly recognized physiological and applied biophysical stimuli impacting macrophage activity, and the potential signalling mechanisms driving downstream responses. The effects of biophysical cues on macrophages’ function in homeostasis and disease and the associated clinical implications are also highlighted.

## Graphical Abstract

**Graphical Abstract:**
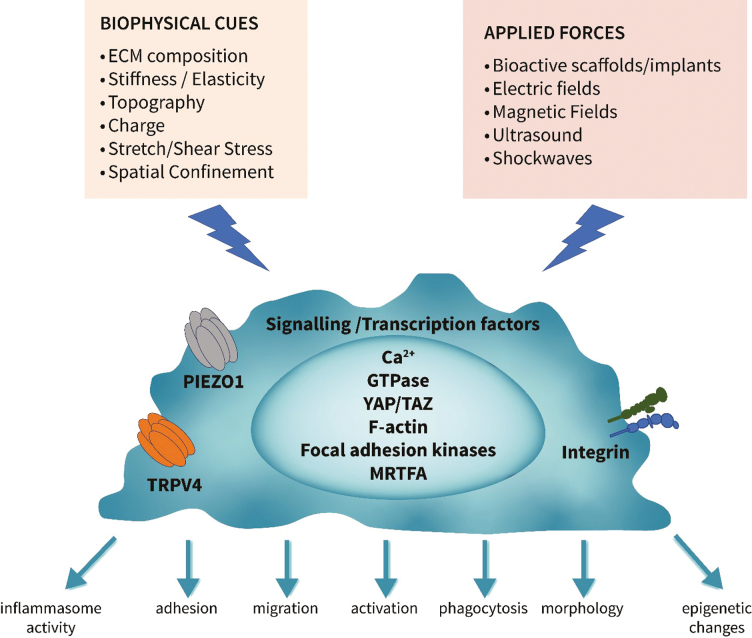

## Introduction

Macrophages are key innate immune cells found in all tissues within the body where they exhibit a plethora of tissue-specific functions, including normal tissue development, maintaining tissue homeostasis, responding to and resolving infection or injury, and metabolic regulation [1]. The phenotype of tissue-resident macrophages is largely directed by the signals from the specific tissue they reside in, where they act as sentinels for the changing environment [2]. Macrophages are also derived from infiltrating blood monocytes that respond to the microenvironment they infiltrate. Macrophages develop functions important in driving inflammation to clear microbes or dead/dying cells and restoring injured tissue back to baseline [3]. The plethora of functions that activated macrophages exhibit is due to their heterogeneous nature. The general concept is that macrophages differentiate into phenotypically and functionally polarized subsets [4–6]. M1-like pro-inflammatory cells are highly glycolytic, fight infection, drive tissue injury, and eliminate tumours through ROS and pro-inflammatory cytokine/chemokine production and enhanced phagocytosis and killing [4]. By contrast, M2-like anti-inflammatory, immunoregulatory cells rely more on oxidative phosphorylation for energy generation, resolve inflammation, repair and remodel injured tissue, clear dying cells, and debris and support tumour growth [4]. This simplified dichotomous M1/M2 notion stemmed from two-dimensional (2D) *in vitro* experiments where macrophages were activated by specific stimuli [5]. Thus, *in vitro* polarized M1 macrophages are induced by stimuli such as microbial products, for example, that ligate toll-like receptors (TLR), or a pro-inflammatory cytokine such as tumour necrosis factor-α (TNF) or interferon-gamma (IFNγ) that induce a strong inflammatory programme. By contrast, M2-like macrophages, polarized *in vitro* by interleukin 4 (IL-4) or IL-13, improve tissue remodelling and repair. However, macrophages *in vivo* will be faced with diverse environmental signals that change in nature and strength over the course of development, ageing, health, and disease [6]. The net result of the environmental stimuli *in vivo* will be a phenotype along a spectrum of activation states, where the conventional *in vitro*-defined M1-like and M2-like activation subsets lie at opposite ends of the spectrum [4, 6]. Innovative three-dimensional (3D) *in vitro* systems are beginning to better recapitulate some of the same changing signals that result in the modification of macrophage properties. Regardless of the classification, macrophages *in vivo* do exhibit signs of polarized pro-inflammatory and anti-inflammatory/immunoregulatory phenotypes and functions, dependant on the integration of the multitude of stimuli they are encountering at any point in time [1]. These *in vivo* polarized macrophages can change their phenotype as the environment changes, and this plasticity is important in restoring tissue homeostasis in a temporal manner.

Macrophages are a key feature in disease, with the number of macrophages infiltrating tissue correlating, in many cases (inflammatory arthritis [7], allograft rejection [8] and cancer [9]) with disease severity, suggesting a pathogenic role [6]. An imbalance of macrophage subsets, for example too many M1-like pro-inflammatory macrophages, is associated with disorders such as atherosclerosis or arthritis, and a disproportion of M2-like macrophages is linked with asthma, fibrosis, or cancer [6]. Therefore, restoring the balance of macrophage subsets or modulation of macrophage activities are being explored as a therapeutic approach for diseases where macrophage function is dysregulated. To manipulate the function of macrophages for therapy, we need to understand the factors that drive their polarization. Traditionally, biological (e.g. microbes), soluble mediators (e.g. cytokines), and chemical stimuli (e.g. drugs), as well as metabolic intermediates and dietary factors, and even physical exercise, circadian rhythms, stress and sex hormones, have been studied as a way of modifying macrophage functions [6]. However, it has become clear that biophysical stimuli and the responses to the physical environment macrophages are exposed to, plays a significant role in the characteristics macrophages develop [10]. In both health and disease, macrophages receive signals from several biophysical aspects of their microenvironment. These cues include the stiffness, topography, and composition of extracellular matrix (ECM) within the tissue architecture; stretch and elasticity, for example, in lung and cardiac tissue; shear force during blood flow; spatial confinement; overall electrostatic charges in surrounding structures and the shape and characteristics of particles being phagocytosed. All of these factors can change dramatically in physiological conditions such as ageing and resolving inflammation, and in pathological conditions, including the changing ECM stiffness and composition in fibrosis, formation of stiff fatty plaques in atherosclerosis, or spatial confinement of cells in cancer [10], This presents another layer of macrophage regulation. Similarly, externally applied physical stimuli and mechanical forces such as electric fields, magnetic fields, shockwaves, pressure therapy, and healthcare biomaterials can all influence macrophage polarization and functional outcome. Macrophages will sense these biophysical stimuli and through the process of mechanotransduction, convert these into intracellular biochemical signals brought about by specific mechanosensitive receptors. These include transient receptor potential (TRP) of vanilloid subtype (TRPV) and Piezo ion channels or through cell adhesion and integrin engagement transduction of mechanical signals [11]. The resultant intracellular signals can synergize with or oppose those induced by biochemical stimuli to modulate gene expression and overall macrophage functions. The types of biophysiological stimuli in the tissue environment and how these changes in pathogenesis, as well as how externally applied physical stimuli can drive changes in macrophage phenotype and function and the mechanisms involved, will be discussed. These are summarized in Table 1.

**Table 1:** Summary of the biophysical and mechanical stimuli, their potential downstream receptors and transcription factors and how this influences the polarisation of macrophages

Stimulus	Potential receptor	Transcription factor	Macrophage polarization/properties	Reference
**ECM composition**				
Fibrin/laminin/vitronectin	Integrins	GTPases	M2	[12, 13]
Collagen I			Increased glycolysis	
**Topography**				
Rougher surfaces	Integrins	YAP/TAZ	M1	[14, 15, 16, 17]
Micropatterning			M2	
Stiffness				
Softer (<0.3 kPa)	Integrins	YAP/TAZ	M2	[18–20, 21–24, 25, 26]
More rigid (>200 kPa)	TRPV4 PIEZO	NFkB	M1, greater migration and phagocytosis	
**Charge**				
Positively charged surface.	PIEZO1?	NFkB?	M1	[27–29, 30]
Zwitterionic surface	Integrins		Increased foreign body response	
**Spatial confinement**	PIEZO1	MRTF-A		[31]
		YAP/TAZ	Decreased M1	
		(HDAC3 levels)		
Stretch			M2	[32, 33, 34, 35, 36]
Cyclic stretch	TRPV4	YAP/TAZ	Decreases M1 & NLRP3 inflammasome	
	PIEZO1			
**Shear stress**	TRPV4	YAP/TAZ	M1	[37]
	PIEZO1			
**Interstitial flow**	Integrins	STAT3/6	M2	[38]
		YAP/TAZ		
**Electric fields**	Integrins	PI3K and ERK	M2	[39–42]
**Magnetic fields**	Integrins	PPARγ	M2	[43–45, 46]
		GTPases		
**Ultrasound**	Integrins	PPARγ, WNT	M2	[47, 48]
**Shockwaves**	Integrins	ERK	M2	[48–52]
		src	Decreased number of macrophages in tissue	

## Impact of biophysical stimuli on macrophage phenotype and function

### Matrix composition and stiffness

The compositional make up of a tissue and the ECM are powerful physiological cues regulating cellular functions of most cells, including macrophages [53]. Macrophages are generally adherent cells, directly contacting their environment, and are therefore very receptive to mechanical stimuli from changes in the tissue matrix composition or stiffness. The ECM composition will differ according to the tissue type or even location within a tissue. The properties macrophages develop will therefore depend on their spatial position. Collagens makes up a large part of the ECM, along with fibronectin, elastin, laminin, tenascin, nidogen, and glycosaminoglycans and their proteoglycans. Macrophages sense collagen morphologies and respond with changes in the expression and activity of metabolism-related proteins [12]. It is well documented that macrophages cultured *in vitro* on distinct matrix substrates, elicit different responses, for example, rate of growth, morphology, and expression of inflammatory mediators, which are different from those cultured directly on tissue culture plastics [12, 54]. Luu TU and co-authors demonstrated a more anti-inflammatory phenotype when macrophages were cultured on laminin and vitronectin, ECM components that proportionally increase in tumours where macrophages contribute to pathology [55]. Specific tissue ECMs also impact macrophage functions [56], with bladder ECM promoting a more pro-inflammatory macrophage gene signature and small intestine ECM driving an anti-inflammatory genotype, although the compositional make-up of the ECM types that could account for the differences was not determined. Fibrosis, characterized by changes in the ratio of ECM molecules, will change the stroma and the way the macrophages interact with the tissue microenvironment, thereby altering macrophage function. For full reviews on the ECM and macrophages and on biochemical interactions between fibrotic stroma and macrophages, we assist readers to refer Refs. [57] and [58].

Tissue stiffness varies considerably in different organs and regions of the body. The ECM in bone is significantly stiffer than that in adipose or brain tissue. For the differences in stiffness of individual tissues, see review [11]. Pathological processes adjust the matrix composition and stiffness of tissue, for example, in fibrosis, cancer, and atherosclerosis. The stiffness of most soft tissues is typically less than 10 kPa, whereas, in diseased states such as fibrosis, stiffness exceeds 20 kPa. Ageing influences tissue stiffness of skin, blood vessels, and bone. Macrophages sense their exposure to a soft or rigid tissue and change their function accordingly. Macrophages grown on stiffer polyacrylamide gel substrates (280 kPa–70 GPa, akin to stiffness of atherosclerotic plaques) proliferate and migrate more efficiently than those softer substrates (1–5 kPa, akin to healthy arteries). This reflects changes in the actin and cytoskeletal activity and the adherence to substrates, respectively [18]. Macrophages simulated by low substrate stiffness (2.55 kPa, commensurate to collagen tissue) presented an enhanced expression of CD86 and production of reactive oxygen species and secreted more IL-1β and TNF-α, as compared to those on greater substrate stiffness (64 kPa, stiffness found in osteoid tissue) [19, 20]. Moreover, macrophages grown on softer substrates (0.3 kPa gels) and activated with TLR agonists, secreted higher levels of TNF-α [20, 59]. However, for the majority of studies, it has been demonstrated that less stiff substrates, for example, adhesion to soft fibrin hydrogels, reduces inflammation when compared to adhesion on rigid substrates and this can be associated with reduced yes-associated protein (YAP) expression and nuclear localization [21–24]. From these conflicting results, it is evident that the properties macrophages develop are not only dependent on the stiffness of the substrate *per se* but also on the composition of materials regulating the stiffness. Culture on glass or tissue culture plastics have a very rigid structure and are significantly more rigid than tissues, whereas hydrogels are generally fabricated to have stiffnesses more in the physiological range. Such properties must be accounted for when comparing effects in published studies with conflicting results, or those trying to extrapolate results from *in vitro* macrophage culture to that of tissue *in vivo*.

The shape macrophages adopt is also influenced by substrate composition and stiffness. Macrophages cultured on more rigid substrates (150 kPa) demonstrate increased filopodial projections and an elongated shape compared to macrophages cultured on less rigid substrates (1.2 kPa) where a rounded morphology is assumed [18, 20]. Macrophage shape is associated with their function, with larger, more rounded macrophages having a more pro-inflammatory phenotype and those that are elongated, or induced to be elongated through micropatterning, exhibit a more anti-inflammatory, pro-resolving phenotype. Thus, the downstream effects of stiffness directing macrophage shape may also relate to the degree of inflammatory functions they adopt [24, 60, 61].

Phagocytosis of microbes or apoptotic cells and debris is a key function of macrophages in tissue remodelling and restoring homeostasis after infection or injury. The overall efficacy of phagocytosis links to the stiffness of the tissue that macrophages reside in. Macrophages cultured on stiff substrates showed greater phagocytosis of non-opsonized and IgG-opsonized latex beads or bacteria compared with lower stiffness [20, 25]. However, others showed no significant effect in bead or bacteria uptake on different substrate stiffnesses [19]. Interestingly, macrophage uptake of oxidized and acetylated low-density lipoproteins and generation of reactive oxygen species were controlled by substrate stiffness [62]. Linking in with these *in vitro* studies, uptake of particles by airway macrophages is impaired in patients with pulmonary fibrosis, where lung stiffness increases [63].

As well as environmental stiffness, the biophysical property of the target influences the phagocytic efficacy. Microbes, dying cells, debris, and cholesterol crystals have very different sizes, shapes, geometry, composition, and stiffness. Soft particles are phagocytosed slower than stiff particles and bone marrow-derived macrophages phagocytosed beads with 20 kPa stiffness six times more effectively than particles of stiffness 3 kPa [64]. This has important implications *in vivo*. For example, as red blood cells age, they become less efficient in their physiological role of regulating the oxygen/carbon dioxide balance in tissues and need to be cleared from circulation. Aged red blood cells are more rigid than younger cells and so are cleared more effectively by the spleen to ensure a healthy, more efficient younger population remains in the circulation [65].

Therefore, the physical environment, through substrate stiffness, contributes significantly to the overall output a macrophage will exhibit. The function the macrophage adopts through this stimulus will, in turn, regulate the physical environment. One example of this is macrophage-induced ECM remodelling via proteases, which in turn would change the ECM biophysical make up.

### Topography, shape, size, and charge

The downstream properties macrophages develop is also impacted by the topography of surfaces they adhere to, with macrophages adhering better to rough surfaces [66]. Our unpublished results revealed that human monocyte-derived macrophages plated on tissue culture transwell inserts, which were of a rougher nature, adhered much more strongly than macrophages cultured on the smooth plastic tissue culture plate under transwell inserts. Moreover, the morphology, spreading, and gene expression of identical macrophage preparations differed between the two matrices. This has been reported by others where micropatterned topography resulted in a different macrophage phenotype to that of macrophages cultured on substrates with no micropatterning [14, 60]. Likewise, rougher titanium surfaces more efficiently induced the expression of pro-inflammatory cytokines and chemokines [15].

Substrate shape strongly influences macrophage phagocytosis with non-spherical or rod-shaped particles being taken up better than spherical particles [67]. The bending stiffness and the composition of prey are of significance in uptake efficacy (see review [68]). The composition of the particle controls uptake efficacy [69] where adhesion to microparticles coated with fibronectin had enhanced uptake by macrophages when compared to vitronectin and fibrinogen coatings. Size of prey is also important, and several studies have shown smaller microbes (e.g. bacteria) are phagocytosed more effectively than large microbes (e.g. yeast) [68]. However, as well as size and shape and composition of the particle to be ingested, the abundance of phagocytic receptors at any point in time must be taken into account when determining phagocytic efficacy [67].

Surface charge and potential simulates a bioelectric microenvironment, which in turn influences macrophage functions. Charged surfaces effectively promote macrophage polarization, and a higher potential intensity was conducive to the upregulation of M2 polarization [27]. Positively charged super-paramagnetic iron oxide nanoparticles had higher uptake and enhanced M1 macrophage polarization more than negatively charged or neutrally charged particles [28]. Furthermore, it has been reported that zwitterionic (containing an equal number of positively and negatively charged functional groups) polyethylene glycol–phosphorylcholine hydrogels, alter the foreign-body response [29].

### Stretch and shear stress

Some types of tissue-specific resident macrophages are constantly exposed to mechanical stretch, such as those in the periodontal ligament during normal mastication, the lung tissue as it expands and contracts, those exposed to gut peristalsis and those in cardiovascular tissue including the beating heart and those squeezing through blood vessels. Stretch as an environmental stimulus drives the polarization of macrophages to an M2-like phenotype and this may relate to them becoming elongated during stretching [32, 33]. Interstitial flow also polarizes macrophages towards an M2-like phenotype via integrin/Src-mediated mechanotransduction pathways involving STAT3/6 [38]. Cyclic stretch negatively regulates IL-1β secretion through the inhibition of NLRP3 inflammasome activation by attenuating the AMP Kinase pathway, while ventilator-induced cyclic stretch of alveolar macrophages activates macrophage NLRP3 inflammasomes [34, 35]. Mechanical strain of macrophages in culture induces proliferation, while cyclic strain suppressed the phagocytosis of macrophages for latex particles [70, 71]. Furthermore, shear stress such as fluid flow in the vasculature or airflow in the lungs can polarize macrophages to a pro-inflammatory phenotype. Shear stress also increases levels of surface proteins involved in inflammatory responses, including expression of metalloproteinases responsible for ECM remodelling and penetration of macrophages between tissues [37].

### Spatial confinement

Tissue resident or infiltrating monocyte-derived macrophages will be spatially confined within individual tissues. This results in close interactions and crosstalk with neighbouring cells and the ECM, and the level of spatial confinement changes macrophage output [72]. The concept of spatial confinement has important consequences for the properties of *in vitro* macrophage culture experiments, where cells are plated at different densities. The size and spreading of macrophages increase after exposure to pro-inflammatory mediators such as LPS. In some elegant studies, it has been demonstrated that preventing macrophage spreading by spatial confinement, suppresses late LPS-activated transcriptional programs by modulating epigenetic alterations (HDAC3 levels and histone 3 lysine 36 dimethylation) [31]. Confinement reduced actin polymerization and LPS-stimulated nuclear translocation of myocardin-related transcription factor A (MRTF-A), thus downregulating pro-inflammatory cytokine secretion (including IL-6, IL-1b, TNF, CXCL9, and iNOS) and phagocytic potential of macrophages. Spatial confinement was also suggested to prevent inflammasome formation [73]. Effects of spatial confinement on macrophage function *in vivo*, where cell number is dynamic and complex, needs more clarification and a fuller analysis will become possible with the advancement of *in situ* spatial multi-omic tissue analysis technologies.

## Applied physical stimuli influencing macrophage function

Many of the studies giving insights into the behaviour of macrophages towards a specific physical stimulus, such as ECM, are based on 2D culture surfaces/substrate materials *in vitro*. Macrophages tend to respond differently in a 3D microenvironment and prospective studies using novel 3D models will more accurately reflect the complicated *in vivo* microenvironments [74]. However, it is clear that these physiological stimuli have a strong potential to impact the function of macrophages residing within tissue and consequently their role in homeostasis and pathology. This knowledge has been exploited to develop synthetic modalities for therapeutic biophysical stimulation targeting macrophages in disease and a selection of these are described below.

### Electric fields

Electric fields occur naturally in the body, for example in wounded tissue where epithelial barriers have been broken [75]. These electric fields accelerate wound healing through directing migration of fibroblasts and keratinocytes. Our work has demonstrated physiological wound strength electric fields also direct the migration of macrophages and significantly enhance phagocytic uptake through clustering of phagocytic receptors that position them for optimum particle uptake [39]. Exposure of macrophages to low-strength electrical fields upregulates PI3K and ERK activation, mobilization of intracellular calcium, and actin polarization to induce effects. A further study used RNA-seq analysis on direct current-treated macrophages and showed that the steroid biosynthesis pathway was affected [40]. *Salmonella* infection generates an electric field in gut epithelium that can influence the directional migration of macrophages [41]. We have also shown the downregulation of T cell activation and polarization by physiological strength electric fields [42]. There are numerous devices that synthetically generate electric fields in tissues, and these have been exploited for wound healing. These devices work, at least in part, by their ability to enhance macrophage migration into wounds, to induce a more healing macrophage polarization phenotype and to augment macrophage uptake of infective microbes, apoptotic cells and debris.

### Magnetic fields

Exposure of macrophages to a non-uniform magnetic field causes macrophage elongation that is associated with M2-like anti-inflammatory macrophages suggesting magnetic fields could be harnessed to reverse inflammation [43]. This dampening of inflammation in macrophages by magnetic fields resulted in significantly decreased levels of IL-6 and IL-10 compared to unexposed counterparts [44]. Similar dampening of inflammation was observed in macrophages prepared from diabetic patients and exposed to magnetic fields. These exposed macrophages showed decreased ROS production, an enhanced M2 anti-inflammatory phenotype, and decreases in IL-1 and IL-6 production, alongside upregulation of collagen type I and integrins. This suggests application of magnetic fields would, for example, improve healing of diabetic ulcers [45].

### Low-intensity pulsed ultrasound and shock waves

Low-intensity pulsed ultrasound is another form of mechanical stimulation that promotes repair. Several studies have shown that low-intensity pulsed ultrasound activates macrophages, enhances mRNA expression of anti-inflammatory genes (Arg-1, PPAR-γ, and IL-4), promotes M2 polarization via the WNT signalling pathway, and inhibits necroptosis via HSP-70 reduction [47, 48]. In muscle, following pulsed ultrasound, the total macrophage population decreased but the proportion of M2-like macrophages increased [48]. Low-intensity shockwave therapy is a similar mode of physical stimulation that also dampens pro-inflammatory responses and influences macrophage functions to promote regeneration and healing [49, 50]. Our own research investigated the role of low-intensity shockwaves on macrophage activity. For this, we studied macrophage content and phenotype in chronic wound punch biopsies from patients with non-healing venous ulcers prior to, and two weeks post-shockwave treatment, and in macrophage cultures treated with clinical shockwave intensities (150–500 impulses, 5 Hz, 0.1 mJ/mm^2^) [51]. Treatment significantly decreased the overall number of macrophages per biopsy area; however, the proportion of M2-like macrophages was increased in keeping with studies using ultrasound. Shockwave treatment of macrophages significantly boosted uptake of apoptotic cells, healing-associated cytokine and growth factors and caused more macrophage elongation, again suggestive of generating a healing macrophage. ERK activity was enhanced in shockwave-treated macrophages, highlighting one mechanotransduction pathway driving downstream gene changes. Shockwave treatment also decreased the overall number of macrophages in other types of tissue [48, 52].

The advantage of using the above physical stimuli for healing and tissue generation is that it is relatively cheap, easily applied, and has little side effects. Moreover, the treatment can be localized to where required. These treatments could, therefore, provide adjuvant therapy for a range of macrophage-mediated diseases. The advantages of each mode of action would have to be weighed up against the ability of these biophysical stimuli to penetrate deep into tissue and reach affected sites and the changes in macrophages they induce at specific strengths.

## Bioactive scaffolds and surgical implants as biophysical stimuli modulating macrophage behaviour

Scaffold- and biomaterial-based immunomodulation has long been used for tissue regeneration, bone repair, or as therapy for failed healing of wounds. Surgical implants such as titanium substrates have been applied for vascular, dental, and orthopaedic purposes to modulate immune responses [76]. An important consideration in biomaterial design is the interplay between the implanted material and host immune system. Biomaterials are traditionally engineered to avoid an inflammatory-based destructive host response, known as the foreign-body reaction. Macrophages play a key role in controlling these foreign body responses through switching their functions, either driving or quelling inflammatory responses and enhancing immunomodulatory effects to the implant to aid the healing process. The 3D structure and organization of bioactive scaffolds provide physical cues that influence macrophage behaviour. As such, the design of scaffolds and implants that minimize macrophage pro-inflammatory, foreign body responses, and maximize healing functions has been the subject of intense research over the years. Rather than prevention of inflammation and/or foreign body responses with manufacturing inert biomaterials, the challenge is to produce material to recruit and guide macrophage responses towards tissue regeneration and healing. The composition, size of particles, shape, charge, porosity, stiffness, structure, and morphology are all important considerations to maximize therapeutic efficacy of scaffolds or implants. How these biophysical properties influence macrophages for this have been outlined above and the reader is also referred to [77]. The biophysical properties of scaffolds are often combined with chemical modification to induce the greatest effects on healing, for example, modified traditional collagen membranes with epigallocatechin-3-gallate achieve better M2 phenotypic macrophage recruitment [78].

## Mechanisms involved in mechanosensing and mechanotransduction in macrophages

The mechanisms by which macrophages receive external physical cues and convert them into intracellular biochemical signals is an important area of interest if we are to further exploit biophysical stimulation for therapeutic gain. Several mechanisms are now recognized, including mechanosensitive ion channels, such as Piezo-Type Mechanosensitive Ion Channel Component 1 (Piezo1) and transient receptor potential vanilloid 4 (TRPV4); and cell adhesion molecules, such as integrins, selectins, and cadherins that lead to Ca^2+^ fluxes, cytoskeletal reorganization, and transcriptional regulation. The molecular basis of mechanotransduction through these will be discussed in the following section.

### Mechanosensitive ion channels

Piezo1 is a non-selective calcium channel expressed in macrophages that conveys mechanical signals from stresses such as stretch. Activation of Piezo1 permits the flow of Ca^2+^ across the membrane to impact macrophage function [79]. Mechanical stimulation of macrophages and monocytes through cyclical hydrostatic pressure triggers an expression program of pro-inflammatory and chemoattractant mediators and this inflammatory mechanosensing response is entirely dependent on Piezo1. Knockout of Piezo1 specifically in myeloid cells modulated their activation, enabling these cells to downregulate their inflammatory potential and enhance wound healing responses [80]. Piezo1 deficiency has also contributed to decreased glycolysis, and consequently decreased pro-inflammatory cytokine production by regulating the expression of Ca^2+^/calmodulin-dependent protein kinase II (CaMKII) and hypoxia-inducible factor1 a (HIF1a) stability, suggesting a link between mechanotransduction and metabolic activation of macrophages [81].

TRPVs respond to mechanical stimuli such as stretch, pressure, and shear stress and are permeable to sodium as well as calcium [82]. Macrophages express TRPV2, TRPV4, TRPC6, and TRPM7 but only TRVP4 is activated through mechanosensing [25]. Activation of TRPV4, like Piezo1, induces a pro-inflammatory profile in macrophages, for example, TRPV4 activation caused pro-inflammatory responses to mechanical ventilation [83]. Macrophages cultured on stiff substrates and activated by LPS showed greater phagocytic activity than those cultured on soft substrates. This change was due to TRPV4, as when TRVP4 was inhibited or expression reduced, no differences were observed [82]. Following *Pseudomonas aeruginosa* infection, TRPV4 enhanced pathogen clearance by macrophages, at least in part through the upregulation of pro-inflammatory pathways [84]. However, TRPV4 activation has also been shown to induce Ca^2+^ influx and MAPK and YAP/transcriptional co-activator with PDZ-binding motif (TAZ) activation and a decrease in pro-inflammatory cytokines and upregulation of IL-10 [82]. TRPV4 activation in response to osmotic or mechanical stress depends on intracellular messengers, including arachidonic acid and PIP_2_ [85]. Once activated, both Piezo1 and TRPV4 downstream signals can integrate with that biochemical signals to regulate macrophage activation and function, examples being activated through TLR4.

### Adhesion molecules

Integrins, focal adhesions, and proteins associated with podosomes play an important role in macrophage mechanosensing to trigger downstream signalling pathways and modify activation, migration, and phagocytosis. Integrins function as mechanosensors by mediating force-induced rearrangements in the cytoskeleton. For example, stiff matrices enhance integrin CD11b-controlled phagocytosis in macrophages [86]. Following stimulation by physical cues, integrin clustering leads to focal adhesions. These sub-cellular structures are signalling hubs regulating the cytoskeleton and consequently immune cell functions through activation of the non-receptor tyrosine kinases, focal adhesion kinase and Src, and downstream RhoA, Rac1, and Cdc42. Macrophages, however, tend to form podosomes rather than focal adhesions post engagement with integrins. Podosomes connect to the intracellular cytoskeleton and are comprised of an actin-rich core surrounded by integrins [87]. We have demonstrated that electric field application to macrophages resulted in polymerized actin and podosomes that become polarized towards the leading, anode-facing edge of electric field-responsive macrophages. This effect related to the electric field enhanced intracellular Ca^2+^ [39]. Integrins can interact with other pathways induced by mechanical stimuli. For example, applying force to integrins indirectly caused activation of TRVP4 resulting in rapid calcium influx through the TRPV4 channel [88].

Other secondary mediators important in connecting mechanotransduction and gene expression are the mechanoresponsive Hippo pathway effectors, YAP, or its homologue, TAZ. YAP can drive pro-inflammatory macrophages on stiff substrates through increased TNF secretion [22]. MRTF-A is another transcriptional regulator altered by mechanical cues. As previously highlighted, spatial confinement of macrophages reduces MRTF-A nuclear localization by downregulating actin polymerization, resulting in downregulation of LPS-induced pro-inflammatory responses [31]. Mitogen-activated protein kinases are associated with Piezo1 and the TRPV4 channel to induce responses [89]. Moreover, the M2-inducing effects of low-intensity pulsed ultrasound are mediated through the WNT pathway by upregulating Frizzled class receptor 5 expression and enhancing β-catenin nuclear translocation in macrophages [48]. There will be integration and coordination with all mechanotransduction signalling pathways, as well as those induced by soluble mediators and chemical cues to dictate the overall function of macrophages at any point in time.

## Clinical implications of biophysical factors on macrophage function

Understanding the biophysical cues that impact macrophages have important implications for the changes that occur due to ageing, the prediction of disease outcome and therapeutic approaches. Ageing disrupts ECM and tissue function and influences its composition, elasticity, and stiffness [57]. Arterial stiffness increases and the density of bone decreases with age. This can impact our macrophage immune responses that are altered with ageing. The inflammatory response that ensues due to tissue injury and/or infection and the remodelling and reparative phases that follow, results in significant changes to the ECM composition, for example, biophysical properties such as stiffness and deposition of thicker and denser collagen fibres in scar tissue [57]. If these changes in the ECM or in scarring do not resolve sufficiently, the effects on macrophages can result in compromised tissue function as observed in disorders such as cancer, atherosclerosis, and fibrosis.

In cancer, the ECM composition will change with corresponding alterations in tissue rigidity [90]. Increased stroma stiffness in clinical breast cancer samples positively correlates with the number of infiltrated tumour-associated macrophages, and this correlation is stronger in more aggressive tumour subtypes [91]. It is likely that expanding cell numbers leads to spatial confinement. The interstitial fluid pressure within the tumour microenvironment increases as a consequence of tumour growth and increased vascular permeability and this will influence macrophage functions. Radiation treatment will also induce physical signals, and all these can change the outcome of macrophage functions within the tumour. Macrophages are well known to guide tumour growth and metastasis through secretion of chemical mediators. Given the impact of the changing biophysical cues in tumours could have on macrophages, future therapeutic regimes could look to change macrophage functions through applying biophysical signals to improve the killing and phagocytic functions of macrophages or alternatively by indirect means through tempering the stiffness and composition of ECM by modulating tissue remodelling enzymes.

Chronic liver diseases result in fibrosis, affecting the liver microenvironment in a manner that influences the properties of infiltrating macrophages or tissue-resident Kupffer cells, thus affecting their functional output and ultimately the timelines of disease. Pulmonary fibrosis is another fibrotic disorder where, over time, the changing lung environment, (overall stretch, stiffness, and composition) significantly influences macrophage function. Tissue-resident airway macrophages in patients with pulmonary fibrosis have impaired phagocytosis compared to age-matched healthy subjects [63]. Other disorders where stiffness will impact disease pathogenesis are in asthma where airway walls stiffen and in ulcerative colitis where intestinal walls become less pliable; both disorders induce macrophage inflammatory responses [57, 92, 93].

In atherosclerosis, changes will occur the arterial wall due to remodelling, including ECM composition and topography, increased stiffness, increased arterial shear stress and development of hypertension. These changes will alter the properties of macrophages infiltrating into plaques [94]. Macrophages will, in turn, further contribute to pathological remodelling. This will influence the stability of the plaque through release of macrophage metalloproteinases and angiogenic factors.

Applied biophysical stimuli have already been used therapeutically to alleviate many disorders and several of these modalities work by potentially exerting effects on macrophages. Extracorporeal shock wave therapy is applied to chronic non-healing wounds when all other treatments fail. We have shown that this treatment has several effects on the healing properties of macrophages [51]. Low-intensity pulsed ultrasound promotes skeletal muscle regeneration via modulating macrophage populations [48]. Moreover, bioelectricity has been implicated in improving wound healing and the strengths applied induce phenotypic changes and important wound healing functions of macrophages. Wound dressings that incorporate bioelectrical cues are being engineered for chronic wounds in patients with severe diseases such as diabetes and lower limb ischemia. Electric fields, shockwaves, and ultrasound have been suggested as an adjuvant therapy for cancer. Although the implications on macrophages in this disorder are not fully understood, from *in vitro* analysis, it is likely that some of the positive effects of this treatment are due to changes in macrophage function.

Designing and engineering scaffolds for bone healing or surgical implants are now taking into account the potential effects they could have on immune cells. Characteristics including their elasticity, pore size, stiffness, topography, composition, and charge are needed for appropriate regulation of macrophage activation and function if healing properties of macrophages are to be maximized. Pharmacologically targeting downstream signalling mediators of physical stimuli, for example, TRVP4, could also impact the function of macrophages [95]. Manipulation of YAP/TAZ has potential as a therapeutic approach for macrophage-mediated pathologies exhibiting changes in their biophysical microenvironment [96, 97].

## Concluding comments

Many questions remain regarding the therapeutic use of biophysical stimuli for immune-mediated diseases. These include the potential long-term effects for scaffolds or implants, which disorders would benefit most from biophysical therapy, how cells other than macrophages are impacted by these cues, and how biochemical cues interact with biochemical signals. A full understanding of the mechanoresponsiveness of macrophages and how their functions can be controlled *in vivo* by biophysical stimuli has its challenges (Reviewed by Adams *et al.* [98]). Regardless of these challenges, it is clear that biophysical forces have a place in the clinic for macrophage-mediated diseases and could provide a more focussed adjuvant treatment with few side effects. Much progress has been made in the field in defining effects of biophysical cues on macrophages *in vitro* and future studies should now focus on the clinical translational potential as a goal.

## Abbreviations:

3D: three-dimensional;
ECM: extracellular matrix;
HDAC: histone deacetylase;
HIF1a: hypoxia-inducible factor1 a;
IF: interstitial flow;
IFNγ: interferon-gamma;
IgG: immunoglobulin G;
IL: interleukin;
MRTF-A: myocardin-related transcription factor A;
NLRP3: NLR family pyrin domain containing 3;
Pa: Pascal;
ROS: reactive oxygen species;
STAT: signal transducer and activator of transcription;
TAZ: transcriptional co-activator with PDZ-binding motif;
TLR: toll-like receptors;
TRPV: transient receptor potential of vanilloid subtype;
TNF: tumour necrosis factor-α;
YAP: yes-associated protein

## References

[1] Wynn TA, Chawla A, Pollard JW. Macrophage biology in development, homeostasis and disease. Nature 2013, 496, 445–55. doi:10.1038/nature12034 23619691 PMC3725458

[2] Davies LC, Jenkins SJ, Allen JE, Taylor PR. Tissue-resident macrophages. Nat Immunol 2013, 14, 986–95. doi:10.1038/ni.2705 24048120 PMC4045180

[3] Serbina NV, Jia T, Hohl TM, Pamer EG. Monocyte-mediated defense against microbial pathogens. Annu Rev Immunol 2008, 26, 421–52. doi:10.1146/annurev.immunol.26.021607.090326 18303997 PMC2921669

[4] Mosser DM, Edwards JP. Exploring the full spectrum of macrophage activation. Nat Rev Immunol 2008, 8, 958–69. doi:10.1038/nri2448 19029990 PMC2724991

[5] Murray PJ, Allen JE, Biswas SK, Fisher EA, Gilroy DW, Goerdt S, et al. Macrophage activation and polarization: nomenclature and experimental guidelines. Immunity 2014, 41, 14–20. doi:10.1016/j.immuni.2014.06.008 25035950 PMC4123412

[6] Murray PJ. Macrophage polarization. Annu Rev Physiol 2017, 79, 541–66. doi:10.1146/annurev-physiol-022516-034339 27813830

[7] Lliso-Ribera G, Humby F, Lewis M, Nerviani A, Mauro D, Rivellese F, et al. Synovial tissue signatures enhance clinical classification and prognostic/treatment response algorithms in early inflammatory arthritis and predict requirement for subsequent biological therapy: results from the pathobiology of early arthritis cohort (PEAC). Ann Rheum Dis 2019, 78, 1642–52. doi:10.1136/annrheumdis-2019-215751 31582377 PMC6900253

[8] Bergler T, Jung B, Bourier F, Kühne L, Banas MC, Rümmele P, et al. Infiltration of macrophages correlates with severity of allograft rejection and outcome in human kidney transplantation. PLoS One 2016, 11, e0156900. doi:10.1371/journal.pone.0156900 27285579 PMC4902310

[9] Binnewies M, Pollack JL, Rudolph J, Dash S, Abushawish M, Lee T, et al. Targeting TREM2 on tumor-associated macrophages enhances immunotherapy. Cell Rep 2021, 37, 109844. doi:10.1016/j.celrep.2021.109844 34686340

[10] Meli VS, Veerasubramanian PK, Atcha H, Reitz Z, Downing TL, Liu WF. Biophysical regulation of macrophages in health and disease. J Leukoc Biol 2019, 106, 283–99. doi:10.1002/JLB.MR0318-126R 30861205 PMC7001617

[11] Lee M, Du H, Winer DA, Clemente-Casares X, Tsai S. Mechanosensing in macrophages and dendritic cells in steady-state and disease. Front Cell Dev Biol 2022, 10, 1044729. doi:10.3389/fcell.2022.1044729 36467420 PMC9712790

[12] Vasse GF, Russo S, Barcaru A, Oun AAA, Dolga AM, van Rijn P, et al. Collagen type I alters the proteomic signature of macrophages in a collagen morphology-dependent manner. Sci Rep 2023, 13, 5670. doi:10.1038/s41598-023-32715-0 37024614 PMC10079972

[13] Seetharaman S, Etienne-Manneville S. Integrin diversity brings specificity in mechanotransduction. Biol Cell 2018, 110, 49–64. doi:10.1111/boc.201700060 29388220

[14] Singh S, Awuah D, Rostam HM, Emes RD, Kandola NK, Onion D, et al. Unbiased analysis of the impact of micropatterned biomaterials on macrophage behavior provides insights beyond predefined polarization states. ACS Biomater Sci Eng 2017, 3, 969–78. doi:10.1021/acsbiomaterials.7b00104 33429569

[15] Tan KS, Qian L, Rosado R, Flood PM, Cooper LF. The role of titanium surface topography on J774A.1 macrophage inflammatory cytokines and nitric oxide production. Biomaterials 2006, 27, 5170–7. doi:10.1016/j.biomaterials.2006.05.002 16808973

[16] Barth KA, Waterfield JD, Brunette DM. The effect of surface roughness on RAW 264.7 macrophage phenotype. J Biomed Mater Res A 2013, 101, 2679–88. doi:10.1002/jbm.a.34562 23427077

[17] Bartneck M, Schulte VA, Paul NE, Diez M, Lensen MC, ZwadloKlarwasser G. Induction of specific macrophage subtypes by defined micro-patterned structures. Acta Biomater 2010, 6, 3864–72. doi:10.1016/j.actbio.2010.04.025 20438871

[18] Adlerz KM, Aranda-Espinoza H, Hayenga HN. Substrate elasticity regulates the behavior of human monocyte-derived macrophages. Eur Biophys J 2016, 45, 301–9. doi:10.1007/s00249-015-1096-8 26613613

[19] Chen M, Zhang Y, Zhou P, Liu X, Zhao H, Zhou X, et al. Substrate stiffness modulates bone marrow-derived macrophage polarization through NF-κB signaling pathway. Bioact Mater 2020, 30, 880–90. doi:10.1016/j.bioactmat.2020.05.004 PMC7332470 32637751

[20] Patel NR, Bole M, Chen C, Hardin CC, Kho AT, Mih J, et al. Cell elasticity determines macrophage function. PLoS One 2012, 7, e41024. doi:10.1371/journal.pone.0041024 23028423 PMC3445606

[21] Previtera ML, Sengupta A. Substrate stiffness regulates proinflammatory mediator production through TLR4 activity in macrophages. PLoS One 2015, 10, e0145813. doi:10.1371/journal.pone.0145813 26710072 PMC4692401

[22] Meli VS, Atcha H, Veerasubramanian PK, Nagalla RR, Luu TU, Chen EY, et al. YAP-mediated mechanotransduction tunes the macrophage inflammatory response. Sci Adv 2020, 6, eabb8471. doi:10.1126/sciadv.abb8471 33277245 PMC7717914

[23] Zhuang Z, Zhang Y, Sun S, Li Q, Chen K, An C, et al. Control of matrix stiffness using methacrylate-gelatin hydrogels for a macrophage-mediated inflammatory response. ACS Biomater Sci Eng 2020, 6, 3091–102. doi:10.1021/acsbiomaterials.0c00295 33463297

[24] Sridharan R, Cavanagh B, Cameron AR, Kelly DJ, O’Brien FJ. Material stiffness influences the polarization state, function and migration mode of macrophages. Acta Biomater 2019, 89, 47–59. doi:10.1016/j.actbio.2019.02.048 30826478

[25] Scheraga RG, Abraham S, Niese KA, Southern BD, Grove LM, Hite RD, et al. TRPV4 mechanosensitive ion channel regulates lipopolysaccharide-stimulated macrophage phagocytosis. J Immunol 2016, 196, 428–36. doi:10.4049/jimmunol.1501688 26597012 PMC4684994

[26] Okamoto T, Takagi Y, Kawamoto E, Park EJ, Usuda H, Wada K, et al. Reduced substrate stiffness promotes M2-like macrophage activation and enhances peroxisome proliferator-activated receptor γ expression. Exp Cell Res 2018, 367, 264–73. doi:10.1016/j.yexcr.2018.04.005 29627321

[27] Wang Z, He X, Tang B, Chen X, Dong L, Cheng K, et al. Polarization behavior of bone marrow-derived macrophages on charged P(VDF-TrFE) coatings. Biomater Sci 2021, 9, 874–81. doi:10.1039/d0bm01604g 33236731

[28] Zhang W, Cao S, Liang S, Tan CH, Luo B, Xu X, et al. Differently charged super-paramagnetic iron oxide nanoparticles preferentially induced M1-like phenotype of macrophages. Front Bioeng Biotechnol 2020, 8, 537. doi:10.3389/fbioe.2020.00537 32548111 PMC7272720

[29] Jansen LE, Amer LD, Chen EY, Nguyen TV, Saleh LS, Emrick T, et al. Zwitterionic PEG-PC hydrogels modulate the foreign body response in a modulus-dependent manner. Biomacromolecules 2018, 19, 2880–8. doi:10.1021/acs.biomac.8b00444 29698603 PMC6190668

[30] Kong Y, Liu F, Ma B, Duan J, Yuan W, Sang Y, et al. Wireless localized electrical stimulation generated by an ultrasound-driven piezoelectric discharge regulates proinflammatory macrophage polarization. Adv Sci (Weinh) 2021, 8, 2100962. doi:10.1002/advs.202100962 34258169 PMC8261497

[31] Jain N, Vogel V. Spatial confinement downsizes the inflammatory response of macrophages. Nat Mater 2018, 17, 1134–44. doi:10.1038/s41563-018-0190-6 30349032 PMC6615903

[32] Matsumoto T, Delafontaine P, Schnetzer KJ, Tong BC, Nerem RM. Effect of uniaxial, cyclic stretch on the morphology of monocytes/macrophages in culture. J Biomech Eng 1996, 118, 420–2. doi:10.1115/1.2796026 8872266

[33] Liang W, Ding P, Qian J, Li G, Lu E, Zhao Z. Polarized M2 macrophages induced by mechanical stretching modulate bone regeneration of the craniofacial suture for midfacial hypoplasia treatment. Cell Tissue Res 2021, 386, 585–603. doi:10.1007/s00441-021-03533-5 34568957

[34] Maruyama K, Sakisaka Y, Suto M, Tada H, Nakamura T, Yamada S, et al. Cyclic stretch negatively regulates IL-1β secretion through the inhibition of NLRP3 inflammasome activation by attenuating the AMP kinase pathway. Front Physiol 2018 Jun, 9, 802. doi:10.3389/fphys.2018.00802 30002631 PMC6031751

[35] Wu J, Yan Z, Schwartz DE, Yu J, Malik AB, Hu G. Activation of NLRP3 inflammasome in alveolar macrophages contributes to mechanical stretch-induced lung inflammation and injury. J Immunol 2013, 190, 3590–9. doi:10.4049/jimmunol.1200860 23436933 PMC3608749

[36] Oya K, Sakamoto N, Ohashi T, Sato M. Combined stimulation with cyclic stretching and hypoxia increases production of matrix metalloproteinase-9 and cytokines by macrophages. Biochem Biophys Res Commun 2011, 412, 678–82. doi:10.1016/j.bbrc.2011.08.024 21867689

[37] Son H, Choi HS, Baek SE, Kim YH, Hur J, Han JH, et al. Shear stress induces monocyte/macrophage-mediated inflammation by upregulating cell-surface expression of heat shock proteins. Biomed Pharmacother 2023, 161, 114566. doi:10.1016/j.biopha.2023.114566 36963359

[38] Li R, Serrano JC, Xing H, Lee TA, Azizgolshani H, Zaman M, et al. Interstitial flow promotes macrophage polarization toward an M2 phenotype. Mol Biol Cell 2018, 29, 1927–40. doi:10.1091/mbc.E18-03-0164 29995595 PMC6232969

[39] Hoare JI, Rajnicek AM, McCaig CD, Barker RN, Wilson HM. Electric fields are novel determinants of human macrophage functions. J Leukoc Biol 2016, 99, 1141–51. doi:10.1189/jlb.3A0815-390R 26718542

[40] Li H, Liu S, Du Y, Tan J, Luo J, Sun Y. Hub proteins involved in RAW 264.7 macrophages exposed to direct current electric field. Int J Mol Sci 2020, 21, 4505. doi:10.3390/ijms21124505 32599940 PMC7352442

[41] Sun Y, Reid B, Ferreira F, Luxardi G, Ma L, Lokken KL, et al. Infection-generated electric field in gut epithelium drives bidirectional migration of macrophages. PLoS Biol 2019, 17, e3000044. doi:10.1371/journal.pbio.3000044 30964858 PMC6456179

[42] Arnold CE, Rajnicek AM, Hoare JI, Pokharel SM, Mccaig CD, Barker RN, et al. Physiological strength electric fields modulate human T cell activation and polarisation. Sci Rep 2019, 9, 17604. doi:10.1038/s41598-019-53898-5 31772211 PMC6879562

[43] Wosik J, Chen W, Qin K, Ghobrial RM, Kubiak JZ, Kloc M. Magnetic field changes macrophage phenotype. Biophys J 2018, 114, 2001–13. doi:10.1016/j.bpj.2018.03.002 29694876 PMC5937143

[44] Ross CL, Harrison BS. Effect of time-varied magnetic field on inflammatory response in macrophage cell line RAW 264.7. Electromagn Biol Med 2013, 32, 59–69. doi:10.3109/15368378.2012.701191 23046146

[45] Zanotti F, Trentini M, Zanolla I, Tiengo E, Mantarro C, Dalla Paola L, et al. Playing with biophysics: how a symphony of different electromagnetic fields acts to reduce the inflammation in diabetic derived cells. Int J Mol Sci 2023, 24, 1754. doi:10.3390/ijms24021754 36675268 PMC9861282

[46] Sun X, Gao Y, Li Z, He J, Wu Y. Magnetic responsive hydroxyapatite scaffold modulated macrophage polarization through PPAR/JAK-STAT signaling and enhanced fatty acid metabolism. Biomaterials 2023, 295, 122051. doi:10.1016/j.biomaterials.2023.122051 36812842

[47] Gouda SAA, Aboulhoda BE, Abdelwahed OM, Abdallah H, Rashed L, Hussein RE, et al. Low-intensity pulsed ultrasound (LIPUS) switched macrophage into M2 phenotype and mitigated necroptosis and increased HSP 70 in gentamicin-induced nephrotoxicity. Life Sci 2023, 314, 121338. doi:10.1016/j.lfs.2022.121338 36592788

[48] Qin H, Luo Z, Sun Y, He Z, Qi B, Chen Y, et al. Low-intensity pulsed ultrasound promotes skeletal muscle regeneration via modulating the inflammatory immune microenvironment. Int J Biol Sci 2023, 19, 1123–45. doi:10.7150/ijbs.79685 36923940 PMC10008697

[49] Davis TA, Stojadinovic A, Anam K, Amare M, Naik S, Peoples GE, et al. Extracorporeal shock wave therapy suppresses the early proinflammatory immune response to a severe cutaneous burn injury. Int Wound J 2009, 6, 11–21. doi:10.1111/j.1742-481x.2008.00540.x 19291111 PMC7951765

[50] Sukubo NG, Tibalt E, Respizzi S, Locati M, d’Agostino MC. Effect of shock waves on macrophages: a possible role in tissue regeneration and remodeling. Int J Surg 2015, 24, 124–30. doi:10.1016/j.ijsu.2015.07.719 26291028

[51] Holsapple JS, Cooper B, Berry SH, Staniszewska A, Dickson BM, Taylor JA, et al. Low intensity shockwave treatment modulates macrophage functions beneficial to healing chronic wounds. Int J Mol Sci 2021, 22, 7844. doi:10.3390/ijms22157844 34360610 PMC8346032

[52] Iwatsu J, Yabe Y, Kanazawa K, Itaya N, Sogi Y, Saijo Y, et al. Extracorporeal shockwave therapy in an immobilized knee model in rats prevents progression of joint contracture. J Orthop Res 2023, 41, 951–61. doi:10.1002/jor.25433 36031592

[53] Vogel V. Unraveling the mechanobiology of extracellular matrix. Annu Rev Physiol 2018, 80, 353–87. doi:10.1146/annurev-physiol-021317-121312 29433414

[54] Armstrong JW, Chapes SK. Effects of extracellular matrix proteins on macrophage differentiation, growth, and function: comparison of liquid and agar culture systems. J Exp Zool 1994, 269, 178–87. doi:10.1002/jez.1402690303 8014614

[55] Luu TU, Liu WF. Regulation of macrophages by extracellular matrix composition and adhesion geometry. Regener Eng Transl Med 2018, 4, 238–46. doi:10.1007/s40883-018-0065-z

[56] Huleihel L, Dziki JL, Bartolacci JG, Rausch T, Scarritt ME, Cramer MC, et al. Macrophage phenotype in response to ECM bioscaffolds. Semin Immunol 2017, 29, 2–13. doi:10.1016/j.smim.2017.04.004 28736160 PMC5612880

[57] Sutherland TE, Dyer DP, Allen JE. The extracellular matrix and the immune system: A mutually dependent relationship. Science 2023, 379, eabp8964. doi:10.1126/science.abp8964 36795835

[58] Vasse GF, Nizamoglu M, Heijink IH, Schlepütz M, van Rijn P, Thomas MJ, et al. Macrophage-stroma interactions in fibrosis: biochemical, biophysical, and cellular perspectives. J Pathol 2021, 254, 344–57. doi:10.1002/path.5632 33506963 PMC8252758

[59] Gruber EJ, Leifer CA. Molecular regulation of TLR signaling in health and disease: mechano-regulation of macrophages and TLR signaling. Innate Immun 2020, 26, 15–25. doi:10.1177/1753425919838322 31955624 PMC6974875

[60] Luu TU, Gott SC, Woo BW, Rao MP, Liu WF. Micro- and nanopatterned topographical cues for regulating macrophage cell shape and phenotype. ACS Appl Mater Interfaces 2015, 7, 28665–72. doi:10.1021/acsami.5b10589 26605491 PMC4797644

[61] McWhorter FY, Wang T, Nguyen P, Chung T, Liu WF. Modulation of macrophage phenotype by cell shape. Proc Natl Acad Sci 2013, 110, 17253–8. doi:10.1073/pnas.1308887110 24101477 PMC3808615

[62] Gruber EJ, Aygun AY, Leifer CA. Macrophage uptake of oxidized and acetylated low-density lipoproteins and generation of reactive oxygen species are regulated by linear stiffness of the growth surface. PLoS One 2021, 16, e0260756. doi:10.1371/journal.pone.0260756 34914760 PMC8675690

[63] Allden SJ, Ogger PP, Ghai P, McErlean P, Hewitt R, Toshner R, et al. The transferrin receptor CD71 delineates functionally distinct airway macrophage subsets during idiopathic pulmonary fibrosis. Am J Respir Crit Care Med 2019, 200, 209–19. doi:10.1164/rccm.201809-1775OC 31051082 PMC6635794

[64] Beningo KA, Wang YL. Fc-receptor-mediated phagocytosis is regulated by mechanical properties of the target. J Cell Sci 2002, 115, 849–56. doi:10.1242/jcs.115.4.849 11865040

[65] Xu Z, Zheng Y, Wang X, Shehata N, Wang C. Stiffness increase of red blood cells during storage. Microsyst Nanoeng 2018, 4, 17103. doi:10.1038/micronano.2017.103

[66] Rich A, Harris AK. Anomalous preferences of cultured macrophages for hydrophobic and roughened substrata. J Cell Sci 1981, 50, 1–7. doi:10.1242/jcs.50.1.1 7033247

[67] Paul D, Achouri S, Yoon YZ, Herre J, Bryant CE, Cicuta P. Phagocytosis dynamics depends on target shape. Biophys J 1143, 105, 38–50. doi:10.1016/j.bpj.2013.07.036 PMC3762343 24010657

[68] Jain N, Moeller J, Vogel V. Mechanobiology of macrophages: how physical factors coregulate macrophage plasticity and phagocytosis. Annu Rev Biomed Eng 2013, 105, 1143–50. doi:10.1146/annurev-bioeng-062117-121224 31167103

[69] Zaveri TD, Lewis JS, Dolgova NV, Clare-Salzler MJ, Keselowsky BG. Integrin-directed modulation of macrophage responses to biomaterials. Biomaterials. 2014, 35(11):3504–15. doi:10.1016/j.biomaterials.2014.01.007 24462356 PMC3970928

[70] Sager HB, Hulsmans M, Lavine KJ, Moreira MB, Heidt T, Courties G, et al. Proliferation and recruitment contribute to myocardial macrophage expansion in chronic heart failure. Circ Res 2016, 119, 853–64. doi:10.1161/CIRCRESAHA.116.309001 27444755 PMC5378496

[71] Miyazaki H, Hayashi K. Effects of cyclic strain on the morphology and phagocytosis of macrophages. BioMed Mater Eng 2001, 11, 301–9. 11790862

[72] Ruder AV, Temmerman L, van Dommelen JMA, Nagenborg J, Lu C, Sluimer JC, et al. Culture density influences the functional phenotype of human macrophages. Front Immunol 2023, 14, 1078591. doi:10.3389/fimmu.2023.1078591 36969194 PMC10036771

[73] Escolano JC, Taubenberger AV, Abuhattum S, Schweitzer C, Farrukh A, Del Campo A, et al. Compliant substrates enhance macrophage cytokine release and NLRP3 inflammasome formation during their pro-inflammatory response. Front Cell Dev Biol 2021, 9, 639815. doi:10.3389/fcell.2021.639815 33855019 PMC8039395

[74] Baker BM, Chen CS. Deconstructing the third dimension: how 3D culture microenvironments alter cellular cues. J Cell Sci 2012, 125, 3015–24. doi:10.1242/jcs.079509 22797912 PMC3434846

[75] Martin-Granados C, McCaig CD. Harnessing the electric spark of life to cure skin wounds. Adv Wound Care (New Rochelle) 2014, 3, 127–38. doi:10.1089/wound.2013.0451 24761353 PMC3928811

[76] Lee S, Choi J, Shin S, Im YM, Song J, Kang SS, et al. Analysis on migration and activation of live macrophages on transparent flat and nanostructured titanium. Acta Biomater 2011, 7, 2337–44. doi:10.1016/j.actbio.2011.01.006 21232636

[77] He T, Xiao Y, Guo Z, Shi Y, Tan Q, Huang Y, et al. Modulation of macrophage function by bioactive wound dressings with an emphasis on extracellular matrix-based scaffolds and nanofibrous composites. Pharmaceutics 2023, 15, 794. doi:10.3390/pharmaceutics15030794 36986655 PMC10053223

[78] Liu Z, Zhu J, Li Z, Liu H, Fu C. Biomaterial scaffolds regulate macrophage activity to accelerate bone regeneration. Front Bioeng Biotechnol 2023, 11, 1140393. doi:10.3389/fbioe.2023.1140393 36815893 PMC9932600

[79] Solis AG, Bielecki P, Steach HR, Sharma L, Harman CCD, Yun S, et al. Mechanosensation of cyclical force by PIEZO1 is essential for innate immunity. Nature 2019, 573, 69–74. doi:10.1038/s41586-019-1485-8 31435009 PMC6939392

[80] Atcha H, Jairaman A, Holt JR, Meli VS, Nagalla RR, Veerasubramanian PK, et al. Mechanically activated ion channel Piezo1 modulates macrophage polarization and stiffness sensing. Nat Commun 2021, 12, 3256. doi:10.1038/s41467-021-23482-5 34059671 PMC8167181

[81] Leng S, Zhang X, Wang S, Qin J, Liu Q, Liu A, et al. Ion channel Piezo1 activation promotes aerobic glycolysis in macrophages. Front Immunol 2022, 13, 976482. doi:10.3389/fimmu.2022.976482 36119083 PMC9479104

[82] Kärki T, Tojkander S. TRPV protein family-from mechanosensing to cancer invasion. Biomolecules 2021, 11, 1019. doi:10.3390/biom11071019 34356643 PMC8301805

[83] Hamanaka K, Jian MY, Townsley MI, King JA, Liedtke W, Weber DS, et al. TRPV4 channels augment macrophage activation and ventilator-induced lung injury. Am J Physiol Lung Cell Mol Physiol 2010, 299, L353–62. doi:10.1152/ajplung.00315.2009 20562229 PMC2951075

[84] Scheraga RG, Abraham S, Grove LM, Southern BD, Crish JF, Perelas A, et al. TRPV4 protects the lung from bacterial pneumonia via mapk molecular pathway switching. J Immunol 2020, 204, 1310–21. doi:10.4049/jimmunol.1901033 31969384 PMC7033031

[85] Michalick L, Kuebler WM. TRPV4-A missing link between mechanosensation and immunity. Front Immunol 2020, 11, 413. doi:10.3389/fimmu.2020.00413 32210976 PMC7076180

[86] Jaumouillé V, Cartagena-Rivera AX, Waterman CM. Coupling of β_2_ integrins to actin by a mechanosensitive molecular clutch drives complement receptor-mediated phagocytosis. Nat Cell Biol 2019, 21, 1357–69. doi:10.1038/s41556-019-0414-2 31659275 PMC6858589

[87] Linder S, Wiesner C. Feel the force: podosomes in mechanosensing. Exp Cell Res 2016, 343, 67–72. doi:10.1016/j.yexcr.2015.11.026 26658516

[88] Matthews BD, Thodeti CK, Tytell JD, Mammoto A, Overby DR, Ingber DE. Ultra-rapid activation of TRPV4 ion channels by mechanical forces applied to cell surface beta1 integrins. Integr Biol (Camb) 2010, 2, 435–42. doi:10.1039/c0ib00034e 20725677 PMC3147167

[89] Blythe NM, Muraki K, Ludlow MJ, Stylianidis V, Gilbert HTJ, Evans EL, et al. Mechanically activated Piezo1 channels of cardiac fibroblasts stimulate p38 mitogen-activated protein kinase activity and interleukin-6 secretion. J Biol Chem 2019, 294, 17395–408. doi:10.1074/jbc.RA119.009167 31586031 PMC6873183

[90] Pickup MW, Mouw JK, Weaver VM. The extracellular matrix modulates the hallmarks of cancer. EMBO Rep 2014, 15, 1243–53. doi:10.15252/embr.201439246 25381661 PMC4264927

[91] Acerbi I, Cassereau L, Dean I, Shi Q, Au A, Park C, et al. Human breast cancer invasion and aggression correlates with ECM stiffening and immune cell infiltration. Integr Biol (Camb) 2015, 7, 1120–34. doi:10.1039/c5ib00040h 25959051 PMC4593730

[92] Cojocaru A, Irvin CG, Haverkamp HC, Bates JH. Computational assessment of airway wall stiffness in vivo in allergically inflamed mouse models of asthma. J Appl Physiol 1985 2008, 104, 1601–10. doi:10.1152/japplphysiol.01207.2007 18420717

[93] Gordon IO, Agrawal N, Willis E, Goldblum JR, Lopez R, Allende D, et al. Fibrosis in ulcerative colitis is directly linked to severity and chronicity of mucosal inflammation. Aliment Pharmacol Ther 2018, 47, 922–39. doi:10.1111/apt.14526 29411405 PMC5842117

[94] Kaess BM, Rong J, Larson MG, Hamburg NM, Vita JA, Levy D, et al. Aortic stiffness, blood pressure progression, and incident hypertension. JAMA 2012, 308, 875–81. doi:10.1001/2012.jama.10503 22948697 PMC3594687

[95] Ludbrook VJ, Hanrott KE, Kreindler JL, Marks-Konczalik JE, Bird NP, Hewens DA, et al. Adaptive study design to assess effect of TRPV4 inhibition in patients with chronic cough. ERJ Open Res 2021, 7, 00269–2021. doi:10.1183/23120541.00269-2021 34350286 PMC8326712

[96] Wang KC, Yeh YT, Nguyen P, Limqueco E, Lopez J, Thorossian S, et al. Flow-dependent YAP/TAZ activities regulate endothelial phenotypes and atherosclerosis. Proc Natl Acad Sci U S A 2016, 113, 11525–30. doi:10.1073/pnas.1613121113 27671657 PMC5068257

[97] Zanconato F, Cordenonsi M, Piccolo S. YAP/TAZ at the roots of cancer. Cancer Cell 2016, 29, 783–803. doi:10.1016/j.ccell.2016.05.005 27300434 PMC6186419

[98] Adams S, Wuescher LM, Worth R, Yildirim-Ayan E. Mechano-immunomodulation: mechanoresponsive changes in macrophage activity and polarization. Ann Biomed Eng 2019, 47, 2213–31. doi:10.1007/s10439-019-02302-4 31218484 PMC7043232

